# Imaging Spectrum and Surveillance of Testicular Adrenal Rest Tumours in Congenital Adrenal Hyperplasia: A Case Series

**DOI:** 10.7759/cureus.108383

**Published:** 2026-05-06

**Authors:** Prathik Sahu, Prerna Agarwal, Venkata Sai, Shriraam Mahadevan

**Affiliations:** 1 Radiology and Imaging Sciences, Sri Ramachandra Institute of Higher Education and Research, Chennai, IND; 2 Radiology, Sri Ramachandra Institute of Higher Education and Research, Chennai, IND; 3 Endocrinology, Sri Ramachandra Institute of Higher Education and Research, Chennai, IND

**Keywords:** congenital adrenal hyperplasia ( cah ), health surveillance, male factor infertility, testicular adrenal rest tumor, testicular lesion, ultrasound (u/s)

## Abstract

Testicular adrenal rest tumours are an important and potentially reversible complication of congenital adrenal hyperplasia caused by chronic adrenocorticotropic hormone stimulation of ectopic adrenal tissue. Delayed diagnosis can result in irreversible testicular damage and infertility, underscoring the need for early detection. We present a case report of three patients with biochemically confirmed congenital adrenal hyperplasia across different age groups who underwent scrotal ultrasound evaluation at a tertiary care centre. A 32-year-old man presenting with infertility demonstrated bilateral mediastinal hypoechoic lesions with internal vascularity and reduced testicular volumes, consistent with advanced Stage III disease. An 18-year-old man showed bilateral early nodular lesions corresponding to Stage II disease with preserved testicular morphology. A nine-year-old boy demonstrated no detectable intratesticular lesions despite biochemical disease, representing preclinical Stage I involvement. These findings illustrate a continuous, age-dependent imaging spectrum of testicular adrenal rest tumours. Scrotal ultrasound is a sensitive and practical modality for early detection, staging, and longitudinal monitoring. Recognition of this progression enables timely intervention and may prevent progression to infertility.

## Introduction

Testicular adrenal rest tumours (TARTs) are benign intra-testicular lesions arising from ectopic adrenal tissue located within the rete testis and are most commonly associated with congenital adrenal hyperplasia (CAH) due to 21-Hydroxylase deficiency [1-3]. These lesions originate from adrenal cortical cells that migrate with the developing gonads during embryogenesis and persist within the testicular parenchyma, retaining responsiveness to adrenocorticotropic hormone stimulation throughout life [2,4].

Chronic elevation of adrenocorticotropic hormone results in progressive hypertrophy and hyperplasia of these ectopic adrenal rest cells, ultimately leading to nodular lesion formation [2,4]. The prevalence of TARTs increases with age and is strongly associated with inadequate hormonal control, with reported rates approaching 40% in adolescents and upto 80% to 90% in adults with CAH [5-7]. The progressive nature of this hormonal stimulation results in gradual and often asymptomatic lesion development, which contributes to delayed clinical detection [7]. This age-dependent increase underscores the importance of long-term surveillance in affected patients, particularly during adolescence and early adulthood when lesion progression is most active.

Although histologically benign, these lesions carry significant clinical relevance due to their impact on testicular architecture and function. Progressive enlargement may result in compression of the seminiferous tubules, leading to impaired spermatogenesis, infertility, and potentially irreversible testicular damage if not detected or managed early [8]. Early identification is therefore essential, particularly as early-stage lesions may demonstrate partial or complete regression with optimised glucocorticoid therapy [8].

Ultrasound is the primary imaging modality for evaluation and follow-up of these lesions due to its high spatial resolution, wide availability, and lack of ionising radiation. The characteristic imaging appearance of bilateral, well-defined hypoechoic nodules adjacent to the mediastinum testis is highly suggestive and allows reliable non-invasive diagnosis in the appropriate clinical context [9-11]. Recognition of these imaging features is essential not only for accurate diagnosis but also to differentiate TARTs from other intratesticular neoplasms, thereby preventing unnecessary surgical intervention [12].

Despite its benign nature, early identification of these lesions remains clinically important, as imaging may provide the earliest indication of disease progression before the onset of overt clinical symptoms [7]. This highlights the role of imaging-based surveillance in at-risk populations with CAH.

Although the natural history of TARTs has been described in larger cohorts, a detailed depiction of the complete imaging spectrum across different age groups within a single study remains limited. This study aims to describe the age-dependent imaging spectrum and clinical progression of TARTs and to emphasise the importance of structured ultrasound-based surveillance in patients with CAH.

## Case presentation

This case series aims to describe the age-dependent imaging spectrum and clinical progression of TARTs in CAH and to highlight the importance of structured ultrasound-based surveillance. Patients were retrospectively identified from our institutional endocrinology and radiology database over the study period. Inclusion criteria comprised male patients with confirmed CAH in whom both testicular and abdominal imaging for adrenal evaluation, along with hormonal levels, were available on follow-up. Patients with incomplete imaging or missing hormonal data were excluded. All eligible CAH patients registered during the study period were reviewed, of whom three met the inclusion criteria and formed the basis of this report. These three patients represented different stages of TART progression, suggesting a possible age-dependent imaging spectrum; however, it is acknowledged that this progression is inferred across three separate patients at distinct disease stages rather than from longitudinal follow-up of a single patient. Given the small sample size and cross-sectional nature of the observations, findings should be interpreted with caution and may not be generalisable, though the cases collectively illustrate a clinically meaningful continuum of TART evolution in CAH.

TARTs were staged according to the ultrasound-based classification system proposed by Claahsen-van der Grinten et al. [2,13], which grades TART severity based on sonographic size, echogenicity, and degree of testicular involvement. Stage I represents small, hyperechoic lesions with no testicular distortion; Stage II indicates larger lesions with partial testicular involvement; and Stage III denotes extensive tumour burden with significant testicular parenchymal replacement and associated risk of obstructive azoospermia. This staging system is widely adopted in the literature for structured surveillance of TARTs in CAH patients.

Case 1

A 32-year-old man with known congenital adrenal hyperplasia presented with primary infertility. Laboratory evaluation revealed elevated adrenocorticotropic hormone, testosterone, and dehydroepiandrosterone sulphate levels.

**Figure 1:**
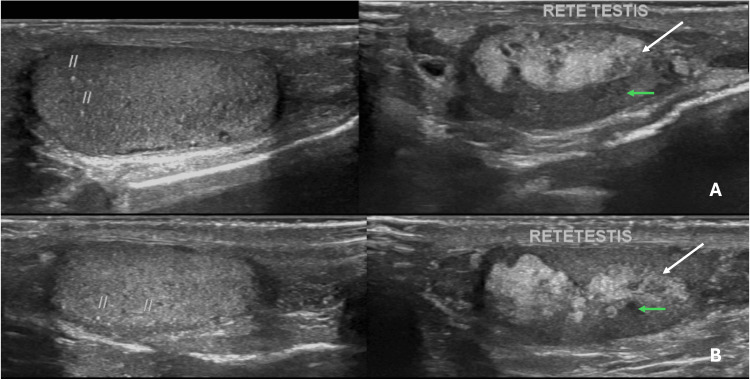
Bilateral scrotal ultrasound demonstrating imaging features of Stage III TARTs. (A) Left testis showing reduced volume with coarse rete testis calcifications (white arrow) and multiple intratesticular microliths (//), with a hypoechoic lesion adjacent to the mediastinum testis (green arrow). (B) Right testis showing similar findings of reduced testicular volume, coarse calcifications (white arrow), intratesticular microliths (//), and a hypoechoic mediastinal lesion (green arrow). Findings are consistent with Stage III disease. TART: testicular adrenal rest tumour

Scrotal ultrasound demonstrated bilaterally reduced testicular volumes, measuring approximately 4.5 mL on the right and 5.1 mL on the left. The testicular parenchyma showed coarse rete testis calcifications with multiple intratesticular microliths. Additionally, bilateral hypoechoic lesions were identified adjacent to the mediastinum testis, demonstrating mild internal vascularity on colour Doppler imaging, consistent with TARTs. These findings are illustrated in Figure 1.

The epididymis was normal bilaterally, with a small incidental cyst in the left epididymis measuring 3 x 3 mm. No hydrocele or extratesticular abnormality was identified.

**Figure 2:**
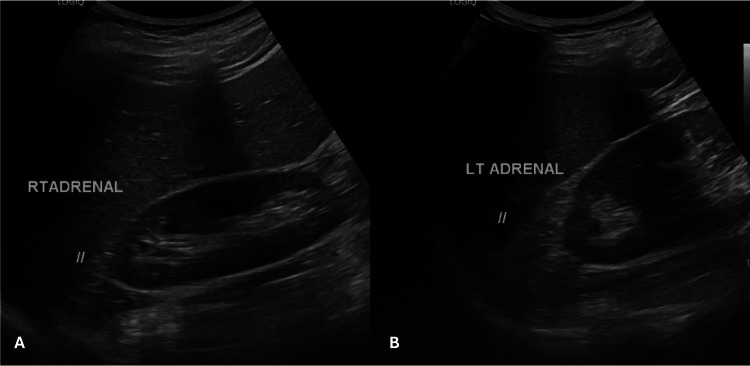
Abdominal ultrasound demonstrating bilateral adrenal enlargement in a patient with CAH, consistent with chronic adrenocorticotropic hormone stimulation. (A) Right adrenal gland showing enlargement (//); (B) Left adrenal gland showing enlargement (//). CAH: congenital adrenal hyperplasia

Correlative abdominal ultrasound demonstrated bilateral adrenal enlargement, as shown in Figure 2. Overall imaging findings were consistent with Stage III disease, with associated testicular volume loss and parenchymal changes.

Case 2

An 18-year-old man with known CAH underwent a screening scrotal ultrasound. Testicular volumes were preserved, measuring approximately 8 mL bilaterally. Multiple punctate intratesticular calcifications consistent with microlithiasis were noted.

**Figure 3:**
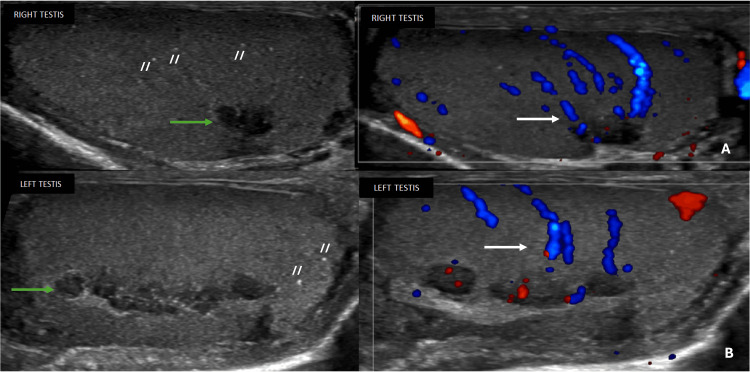
Scrotal ultrasound demonstrating early-stage (Stage II) TART with preserved testicular architecture. (A) Right testis showing preserved volume with multiple punctate intratesticular calcifications- microlithiasis (//) without diffuse parenchymal distortion. A small irregular hypoechoic lesion (green arrow) is noted adjacent to the mediastinum testis with minimal internal and peripheral vascularity on Doppler imaging (white arrow). (B) Left testis showing similar findings of preserved volume with intratesticular microlithiasis (//) and small hypoechoic mediastinal lesion (green arrow) with minimal vascularity (white arrow). No mass effect or vascular compression is seen. TART: testicular adrenal rest tumour

These findings are illustrated in Figure 3. Additionally, bilateral irregular hypoechoic lesions were identified adjacent to the mediastinum testis, more prominent on the right, measuring approximately 13 x 3 mm on the right and 16 × 2 mm on the left. These lesions demonstrated minimal internal and peripheral vascularity without vascular distortion or mass effect, consistent with early-stage TARTs. The epididymis, spermatic cord, and pampiniform plexus were unremarkable, with no evidence of hydrocele or extratesticular pathology. Overall findings were consistent with Stage II disease.

Case 3

**Figure 4:**
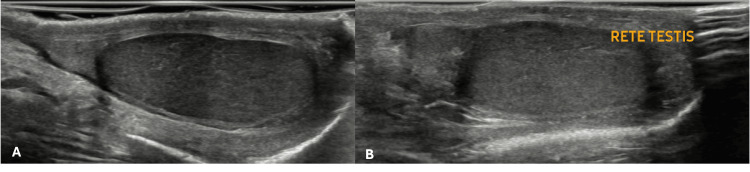
Scrotal ultrasound in a paediatric patient with biochemical evidence of CAH demonstrating normal testicular morphology, consistent with preclinical (Stage I) disease. (A) Right testis showing normal size and homogeneous echotexture without focal intratesticular lesions. (B) Left testis showing normal size and echotexture without focal intratesticular lesions. CAH: congenital adrenal hyperplasia

A nine-year-old boy with biochemical evidence of CAH underwent screening ultrasound evaluation. Scrotal ultrasound demonstrated normal testicular size and echotexture, with no focal intratesticular lesions identified. These findings are illustrated in Figure 4.

**Figure 5:**
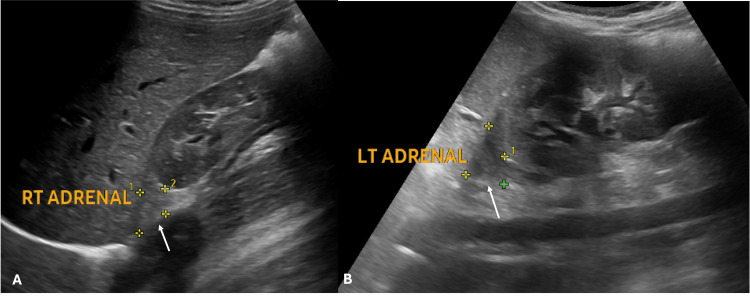
Abdominal ultrasound demonstrating bilateral adrenal hyperplasia (arrow) in a paediatric patient with congenital adrenal hyperplasia, with no detectable testicular lesions (A) Right adrenal gland showing hyperplasia (arrow). (B) Left adrenal gland showing hyperplasia (arrow).

Abdominal ultrasound revealed bilateral adrenal hyperplasia, as shown in Figure 5. In the clinical context, these findings represent preclinical Stage I disease. The hormonal profile of patients with CAH demonstrating stage-wise progression of TARTs is summarised in Table 1.

**Table 1:** Hormonal profile of patients with congenital adrenal hyperplasia across stages of TARTs Table 1 represents an inferred spectrum of TART progression across three patients at different disease stages; this does not represent documented longitudinal progression in a single patient. ª LH reference ranges are age-dependent; a prepubertal reference range of <0.3 mIU/mL applies to Case 3 (9 years), while an adult male reference range of 1.7-8.6 mIU/mL applies to Cases 1 and 2 (32 and 18 years, respectively). The elevated LH of 1.26 mIU/mL in Case 3 may reflect early hypothalamic-pituitary axis activation in the context of poorly controlled CAH. ᵇ Testosterone in Case 1 (32 years) measured 480 ng/dL, which falls within the adult male reference range (300–1000 ng/dL). This value is not elevated in isolation but should be interpreted in the context of concomitantly elevated adrenal androgens, suggesting partial suppression of gonadotropin-driven testicular testosterone production. ᶜ The androstenedione value of 29,200 ng/dL in Case 1 (32 years) was verified from the original laboratory report. This extreme elevation is consistent with severe, longstanding poorly controlled CAH with significant adrenal androgen excess. The hormonal parameters presented reflect cross-sectional values from three separate patients at distinct disease stages and do not represent longitudinal follow-up data from a single patient. All hormonal parameters presented were obtained at the time of, or within four weeks of, the corresponding ultrasound examination, ensuring clinico-radiological correlation. Abbreviations: TART, testicular adrenal rest tumour; LH, luteinizing hormone; ACTH, adrenocorticotropic hormone; 17-OHP, 17-hydroxyprogesterone; DHEAS, dehydroepiandrosterone sulfate; CAH, congenital adrenal hyperplasia

Parameter	Normal Reference Range	Case 1 (32 years, Stage III)	Case 2 (18 years, Stage II)	Case 3 (9 years, Stage I)	Units
LH	<0.3 (prepubertal) / 1.7–8.6 (adult male)	0.4	0.6	1.26	mIU/mL
ACTH	10–60	1800	600	177	pg/mL
17-OHP	<2 (prepubertal) / <5 (adult)	286	110	56	ng/mL
Serum cortisol	5–25	1.2	2.5	4	µg/dL
Testosterone	≤36 (prepubertal) / 300–1000 (adult male)	480	320	96.8	ng/dL
DHEAS	35–430	2600	1200	296	µg/dL
Progesterone	0.2–1.0	3.5	2.1	17.5	ng/mL
Androstenedione	30–200	29,200	450	200	ng/dL

**Figure 6:**
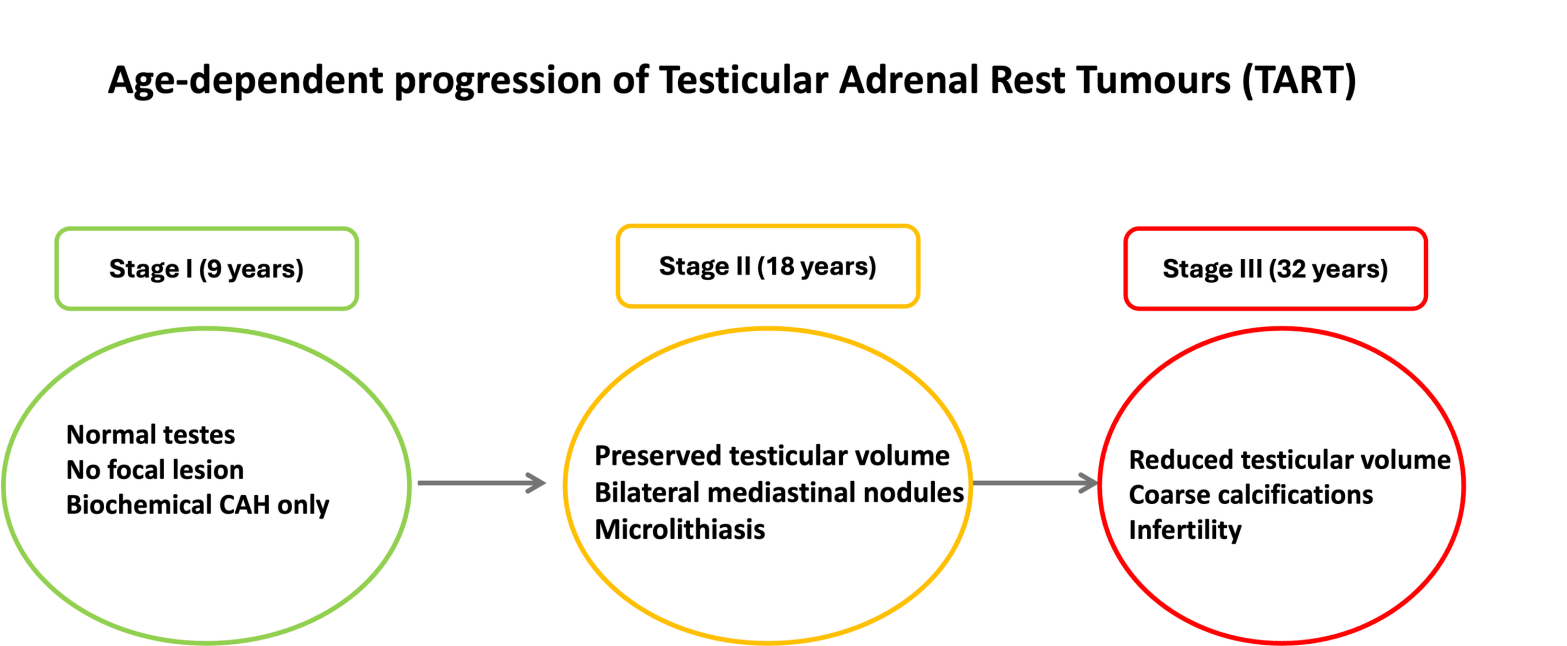
Illustration of the age-related progression spectrum of TARTs in our study Created using Microsoft PowerPoint (non-AI-generated schematic). Illustration of the inferred age-related progression spectrum of TARTs across three patients at distinct disease stages in CAH. It is important to note that this figure represents a cross-sectional inference of disease progression observed across three separate patients: a nine-year-old (Stage I), an 18-year-old (Stage II), and a 32-year-old (Stage III). It does not depict documented longitudinal progression in a single patient over time. The staging corresponds to the ultrasound-based classification described by Claahsen-van der Grinten et al [13]. The spectrum is presented to illustrate the clinically meaningful continuum of TART evolution with advancing age and duration of poorly controlled CAH, and should be interpreted with caution given the small sample size and cross-sectional nature of the observations. TART: testicular adrenal rest tumour; CAH: congenital adrenal hyperplasia

A clinical timeline illustrating the age-dependent progression of TARTs is depicted in Figure 6. The age-wise clinical presentation along with hormonal profile, imaging findings, and staging of patients with CAH is summarised in Table 2.

**Table 2:** Age-wise presentation, hormonal profile, imaging findings, and staging in patients with CAH CAH: congenital adrenal hyperplasia; ACTH: adrenocorticotropic hormone; 17-OHP: 17-hydroxyprogesterone

Age	Presentation	Hormonal Profile	Imaging Findings	Stage
32	Infertility	ACTH ↑, Testosterone ↑	Bilateral nodules	Stage III
18	Known CAH	ACTH ↑	Small nodules	Stage II
9	Screening	17-OHP ↑	Normal	Stage I

## Discussion

**Figure 7:**
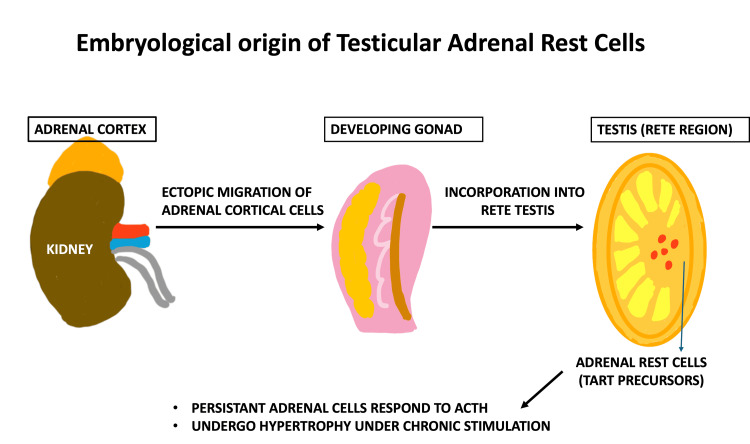
Schematic illustration of the embryological origin and pathogenesis of TARTs in CAH. The figure was created by the authors using hand-drawn elements and Microsoft PowerPoint. TART, testicular adrenal rest tumour; CAH, congenital adrenal hyperplasia; ACTH, adrenocorticotropic hormone

TARTs are a well-recognised complication in adult male patients with CAH, with a reported prevalence of up to 94% [1]. They arise from ectopic adrenal cells within the rete testis that retain functional responsiveness to adrenocorticotropic hormone, leading to progressive hypertrophy and nodular transformation under conditions of chronic hormonal stimulation [1-4], as illustrated in Figure 7.

These tumours are benign lesions that are typically bilateral and are located in the region of the rete testis. On histological examination, they demonstrate features closely resembling adrenal cortical tissue. They are non-encapsulated and composed of interlacing cords and sheets of large polygonal cells with abundant eosinophilic cytoplasm. From a diagnostic standpoint, distinguishing TART from Leydig cell tumours can be challenging based on morphology alone. However, key differentiating features exist: TARTs are bilateral in the majority of cases, whereas Leydig cell tumours are usually unilateral, with bilateral involvement being uncommon. In addition, Reinke crystals, which may be present in a subset of Leydig cell tumours, are absent in TART. Importantly, while a small proportion of Leydig cell tumours may undergo malignant transformation, no cases of malignant behaviour have been reported in TART [14,15].

The clinical significance of these lesions lies not in their benign histology but in their progressive impact on testicular architecture and fertility. Compression of seminiferous tubules and peri-tubular fibrosis can result in irreversible spermatogenic failure if diagnosis is delayed [8,9]. This highlights the importance of early detection, particularly in adolescent and young adult patients, where intervention may still prevent progression.

In contemporary practice, severe forms of CAH can often be identified early through neonatal screening programs or prenatal diagnosis in affected families, thereby reducing the risk of acute life-threatening presentations. Despite early detection in many cases, long-term complications remain relevant in adult patients with CAH, and several of these manifestations may already be present during childhood. Among male patients, one of the most clinically significant complications is the development of testicular masses, including TART [2-4].

In the present study, we demonstrate a clear age-dependent imaging spectrum of TARTs across three patients with CAH. The paediatric patient showed no detectable focal lesions despite biochemical disease, consistent with a preclinical stage. The adolescent patient demonstrated early bilateral mediastinal nodules with preserved testicular volume, while the adult patient exhibited advanced disease with reduced testicular volume and associated infertility. This progression supports the concept that TART evolves along a continuous spectrum rather than appearing as discrete static lesions.

Ultrasound remains the primary imaging modality for evaluation due to its high spatial resolution, accessibility, and ability to detect early mediastinal lesions. The characteristic appearance of bilateral, hypoechoic, mediastinal-based nodules with variable vascularity is highly suggestive in the appropriate clinical context [9-11]. Doppler evaluation may further aid in the differentiation of TARTs from other intratesticular pathologies; TARTs characteristically demonstrate low-grade vascularity without the chaotic, high-velocity flow patterns more commonly associated with malignant neoplasms [15]. However, it should be acknowledged that vascularity patterns alone are not sufficient to reliably exclude malignancy, as overlap exists between benign and malignant lesions on Doppler imaging. Doppler findings should therefore always be interpreted in conjunction with greyscale sonographic features, clinical context, biochemical parameters, and where necessary, tumour markers such as AFP and β-hCG, to arrive at a confident diagnosis and avoid unnecessary surgical intervention. 

Differentiating TART from other intratesticular lesions remains essential. Leydig cell tumours are typically unilateral and not confined to the mediastinum, while seminomas present as homogeneous hypoechoic masses without the characteristic bilateral mediastinal distribution seen in adrenal rest tumours [16,17]. Recognition of these distinguishing imaging features helps avoid unnecessary orchiectomy and guides appropriate medical management.

Hormonal control plays a central role in both prevention and potential regression of early-stage lesions. Adequate glucocorticoid therapy can reduce adrenocorticotropic hormone stimulation, which may stabilise or partially regress early tumours [18]. However, advanced lesions associated with fibrosis are less likely to regress, reinforcing the need for early surveillance.

Although larger studies have described the prevalence and imaging features of TART, there is limited literature demonstrating a complete spectrum of disease progression across different age groups within a single cohort. The present study adds value by illustrating this continuum from preclinical to advanced disease within a structured imaging framework, reinforcing the importance of longitudinal surveillance.

The findings of this case series are consistent with and further reinforce the existing guideline recommendation for periodic testicular ultrasound screening in all males with classic CAH. The Endocrine Society Clinical Practice Guideline (Recommendation 6.13) explicitly advocates periodic testicular ultrasound to assess for the development of TARTs in males with classic CAH, graded as a strong recommendation [19]. Furthermore, current expert opinion suggests that screening should commence in adolescence or upon completion of puberty, given the progressive nature of TART development with advancing age and duration of poorly controlled disease [19]. Ultrasound remains the preferred initial surveillance modality owing to its wide availability, lower cost, and absence of radiation burden. While the small sample size of the present series precludes definitive conclusions, our observations reinforce the clinical value of structured ultrasound-based surveillance in facilitating early TART detection, monitoring disease progression, and guiding timely optimisation of glucocorticoid therapy [20]. Early identification allows timely endocrine optimisation and may preserve fertility potential, which is often irreversibly compromised in advanced disease [19]. Ultrasonography, therefore, represents a cost-effective and highly reliable first-line imaging modality for the screening and detection of TARTs in patients with CAH [21].

## Conclusions

TARTs represent an important and potentially preventable cause of infertility in patients with CAH, demonstrating a characteristic age-dependent progression from preclinical disease to advanced intratesticular involvement. It is acknowledged, however, that this progression is inferred across three patients at distinct disease stages rather than from longitudinal follow-up within the present cohort, and findings should therefore be interpreted with caution.

Ultrasound serves as a key imaging modality for early detection and staging of TARTs, enabling identification of subtle mediastinal lesions in early-stage disease and parenchymal damage in advanced stages. While longitudinal follow-up data were not available in the present series, the cross-sectional imaging findings across the three patients collectively illustrate the sonographic spectrum of TART evolution, underscoring the potential value of structured ultrasound-based surveillance in monitoring disease progression over time.

Recognition of this imaging spectrum is crucial, as timely diagnosis and optimised hormonal control may help prevent irreversible testicular damage. In accordance with established guidlines, periodic testicular ultrasound is strongly recommended in all males with classic CAH, with surveillance commencing at adolescence or upon completion of puberty and continuing annually thereafter. The implementation of such structured surveillance protocols is essential for appropriate management and preservation of fertility outcomes in affected patients.
